# Methanol-induced optic neuropathy: a still-present problem

**DOI:** 10.1007/s00204-021-03202-0

**Published:** 2022-01-06

**Authors:** Sławomir Liberski, Bartlomiej J. Kaluzny, Jarosław Kocięcki

**Affiliations:** 1 Department of Ophthalmology, Poznan University of Medical Sciences, ul. Augustyna Szamarzewskiego 84, 61-848 Poznań, Poland; 2 Division of Ophthalmology and Optometry, Department of Ophthalmology, Collegium Medicum, Nicolaus Copernicus University, ul. Kornela Ujejskiego 75, 85-168 Bydgoszcz, Poland

**Keywords:** Methanol, Methyl alcohol, Toxic optic neuropathy, Methanol-induced optic neuropathy, Optic nerve, Ocular toxicology

## Abstract

Methanol-induced optic neuropathy (Me-ION) is a serious condition that may result in long-term or irreversible visual impairment or even blindness secondary to damage and loss of function of the optic nerve and retina. Me-ION shows a tendency to occur as mass poisonings around the world with a clear predilection for poor societies in developing countries. The main mechanism underlying the molecular basis of Me-ION is the inhibition of the mitochondrial oxidative phosphorylation process through the binding of the toxic metabolite of methanol—formic acid—with the key enzyme of this process—cytochrome c oxidase. However, other mechanisms, including damage to the eye tissues by oxidative stress causing the intensification of the oxidative peroxidation process with the formation of cytotoxic compounds, as well as an increase in the synthesis of pro-inflammatory cytokines and influence on the expression of key proteins responsible for maintaining cell homeostasis, also play an important role in the pathogenesis of Me-ION. Histopathological changes in the eye tissues are mainly manifested as the degeneration of axons and glial cells of the optic nerve, often with accompanying damage of the retina that may involve all its layers. Despite the development of therapeutic approaches, persistent visual sequelae are seen in 30–40% of survivors. Thus, Me-ION continues to be an important problem for healthcare systems worldwide.

## Introduction

Methanol poisoning is a serious life-threatening condition with a mortality rate ranging from 18 to 44% (Noor et al. 2020). Annually in the US, about 5.000 cases are diagnosed with an incidence of 6.4 cases per 1 million hospitalizations, and with the mean age of patients being 38 ± 18 years with a distinct predominance of the male sex (70 vs. 30%) (Kaewput et al. 2021; Kraut 2016). According to statistical data, the prevalence of methanol intoxication is most common in developing countries and Southeast Asia, mainly among the lower economic and social strata (Abrishami et al. 2011; Noor et al. 2020), and is usually associated with accidental oral consumption due to similar physical properties to ethanol, especially in countries with alcohol prohibition, where a higher percentage of methanol poisonings is observed secondary to the oral consumption of contaminated alcohol from illegal domestic production (Khalili et al. 2021). In the US, unintentional consumption of methanol is the cause of 90% of poisonings, while among cases involving intentional ingestion, suicide attempts slightly predominate (Chung et al. 2018). Another important circumstance is occupational exposure—cases of chronic percutaneous (İşcan et al. 2013) and inhalational (Ma et al. 2019; McCormick et al. 1990; Zhao et al. 2015) exposure to methanol leading to clinically significant consequences have been well documented.

Poisoning with methanol and the product of its metabolism—formic acid—is not harmful to non-primates. However, in primates, including humans, the toxic effect due to ineffective metabolism of formic acid is present (Eells et al. 1981; Fu et al. 2017). The first report of methanol toxicity to the visual system was published by MacFarlan in 1855 (MacFarlan 1855). Methanol-induced optic neuropathy (Me-ION) is a serious condition that can result in long-term or even irreversible visual impairment secondary to damage and loss of function of the optic nerve; however, in many cases, a simultaneous disturbance of other structures—the retina, as well as chiasms and the optic tract is present (Grzybowski et al. 2015). According to estimates, visual symptoms occur in about 50% of the cases of methanol poisoning (Grzybowski et al. 2015; Klein et al. 2017; Seme et al. 2001). The exact dose causing pathological changes in the human visual system have not been precisely determined, and in most cases, it may depend on individual metabolic predisposition; however, it is known that consuming as little as 4 ml of methanol can lead to complete loss of vision (Bennett et al. 1953). Massive outbreaks of toxic optic neuropathy (TON) associated with acute or chronic methanol poisoning occurred in the last century in the 1950s in the US (Bennett et al. 1953; Benton and Calhoun 1953) and in the 1990s in Cuba (Lincoff et al. 1993; Sadun et al. 1994), as well as in recent decades in the El Salvador (Hassanian-Moghaddam et al. 2015), Norway (Hovda et al. 2005), Estonia (Paasma et al. 2007), Czech Republic (Zakharov et al. 2014), Libya (Rostrup et al. 2016), Kenya (Rostrup et al. 2016), Uganda (Doreen et al. 2020), Turkey (Gulen et al. 2020), India (Kumar et al. 2019), and Tunisia (Brahmi et al. 2007). Furthermore, in recent months, changes in social functioning caused by the COVID-19 pandemic have also contributed to a significant increase in the incidence of optic neuropathy related to methanol poisoning reported in various regions of the world including Iran (Khalili et al. 2021; Sefidbakht et al. 2020), as well as the US (Yip et al. 2020).

Despite the development of diagnostic methods, prompt diagnosis, and implementation of appropriate treatment, Me-ION is still a challenge for clinicians due to its severe health consequences that significantly reduce the quality of life of survivors many years after discharge (Rulisek et al. 2020), as well as the economic burden on health systems (Barták et al. 2021)—this issue continues to be a major health care problem worldwide. In this review, we attempt to analyze and discuss the current state of knowledge regarding the pathophysiology, clinical presentation, diagnosis, treatment, as well as prognosis for Me-ION.

### Systemic effects of methanol intoxication

Primates, including humans, in contrast to non-primates, are sensitive to methanol due to the limited ability to rapidly metabolize and eliminate methanol and its metabolites from the body (Eells et al. 2000; Plaziac et al. 2003). Formic acid inhibits oxidative phosphorylation by binding to a key enzyme in the mitochondrial respiratory chain—cytochrome c oxidase—causing intracellular adenosine triphosphate (ATP) deficiency (Ahiskali et al. 2019; Saoudi et al. 2011). This effect was observed in both in vitro and in vivo studies at the concentration of formic acid ranging from 5 to 30 nM (Treichel et al. 2003). Disruption in mitochondrial electron transport and production of ATP results in the intensification of anaerobic metabolism with a secondary accumulation of formate and lactate in tissues and the development of metabolic acidosis with secondary serious systemic complications (Gabay et al. 2018; Treichel et al. 2003). Due to the high molar concentration, methanol causes a marked serum osmolal gap, while the anion gap and metabolic acidosis result from the accumulation of formate and lactate in the serum (Hovda et al. 2004). Importantly, as blood pH decreases, the concentration of non-ionized methanol increases, which determines its increased penetration into tissues and a more extensive range of their damage (Garner et al. 1995). Moreover, animal studies have shown that despite almost complete metabolism and removal of methanol from the body, the concentration of formic acid simultaneously tends to persist, causing further tissue damage and delayed regeneration of retinal function (Liu et al. 2016).

### Pharmacokinetics of methanol in humans

**Fig. 1:**
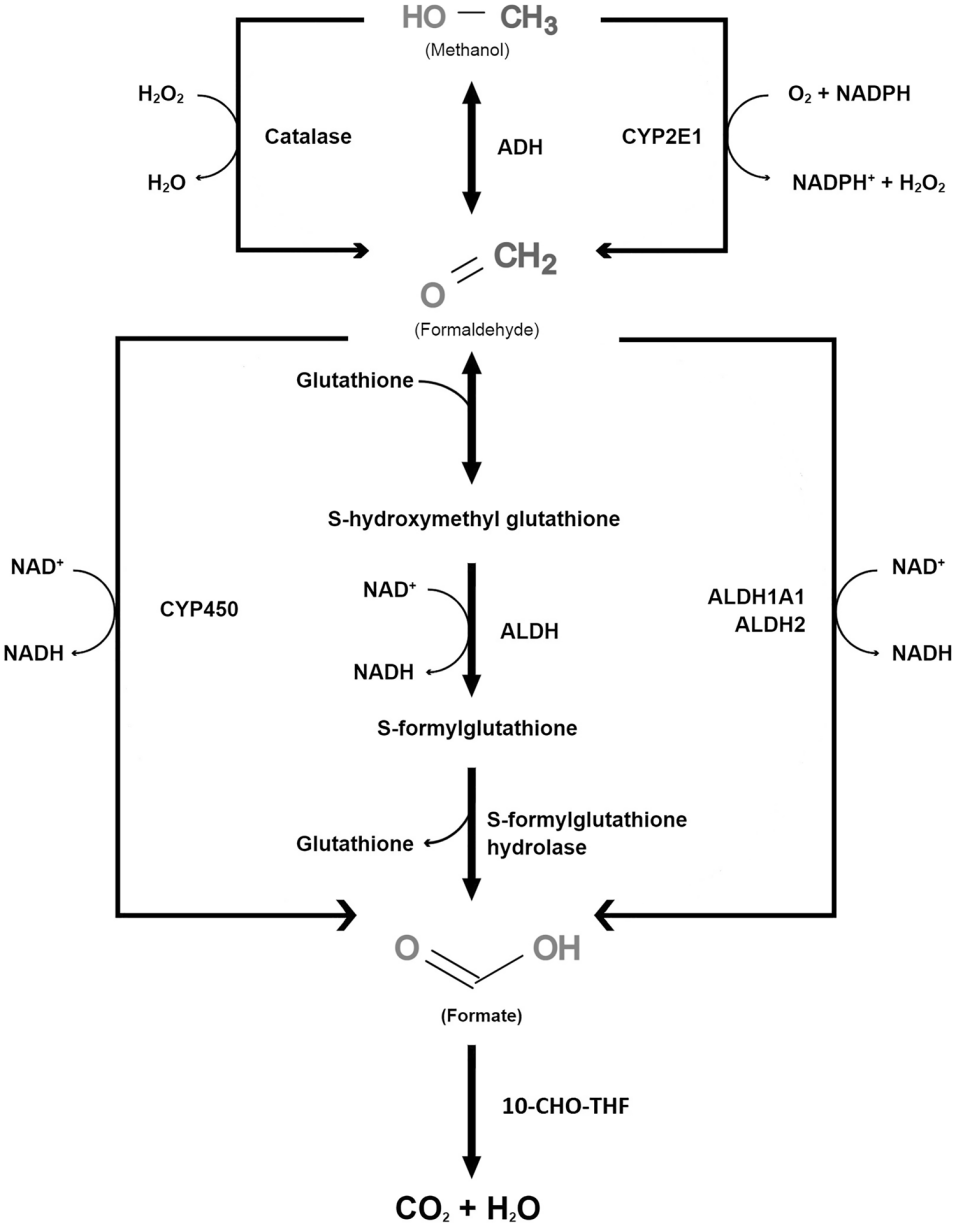
Metabolism of methanol and its metabolites in the human body. Abbreviations: *10-CHO-THF* 10-formyltetrahydrofolate, *ADH* alcohol dehydrogenase, *ALDH* aldehyde dehydrogenase, *ALDH1A1* aldehyde dehydrogenase 1 family, member 1, *ALDH2* mitochondrial aldehyde dehydrogenase, *CYP2E1* cytochrome P450 Family 2 Subfamily E Member 1, *NAD* nicotinamide adenine dinucleotide, *NADH* reduced form of nicotinamide adenine dinucleotide, *NADPH* nicotinamide adenine dinucleotide phosphate

It is known that methanol, compared to its metabolites, has much lower toxic properties toward animal cells (Dorokhov et al. 2015), and a low concentration of methanol (0.12–3.86 µg/mL) can be detected in the plasma of healthy people without optic neuropathy (Dorokhov et al. 2015; Hayasaka et al. 2000). After ingestion, methanol is easily distributed in the body—its presence in the blood, urine, cerebrospinal fluid (CSF), as well as in the vitreous and aqueous humor during a post-mortem examination of poisoned victims was detected (Benton and Calhoun 1953). Due to its rapid absorption by the oral mucosa and further parts of the gastrointestinal tract, methanol reaches its maximum concentration in the serum after 30–90 min (Abrishami et al. 2011; Liu et al. 2016). Then, methanol undergoes slow liver metabolism to its toxic metabolites: formaldehyde, the intermediate compound with a serum half-life of about 1 min, and the more stable, and toxic formic acid. It can be excreted in the urine or become decomposed into water and carbon dioxide via the tetrahydrofolate-dependent pathway (Fig. 1). These reactions are catalyzed by specific enzymes—alcohol dehydrogenase 1b (ADH1b), aldehyde dehydrogenase (ALDH), and N-10-formyltetrahydrofolate dehydrogenase (10-CHO-THF), respectively (González-Quevedo et al. 2002; Liu et al. 2016). There are two more specific pathways of exogenous methanol metabolism, the first one involving monooxygenase cytochrome P450 Family 2 Subfamily E Member 1 (CYP2E1), the activity of which is particularly expressed at high methanol concentrations in tissues, while the second pathway involves the enzyme catalase-H_2_O_2_ (Dorokhov et al. 2015). The last two pathways are responsible for 9% and 1% of methanol metabolism in the human body, respectively; however, it is postulated that their role is dominant within the central nervous system (CNS) (Dorokhov et al. 2015). Compared to ethanol—a similar compound—methanol is eliminated from the body about 6.5 times slower, which facilitates its accumulation in the body (Ingemansson 1984). According to the results of current studies, 70–97% of methanol is excreted from the body after metabolism, while only a small percentage is excreted unchanged in the exhaled air and with the urine (Dorokhov et al. 2015).

### Systemic manifestation of methanol intoxication in humans

The classic triad of systemic symptoms occurring in the course of methanol includes CNS depression, metabolic disorders manifested mainly as systemic metabolic acidosis, and visual disturbances (Gabay et al. 2018; Sadun 1998). As a consequence of the slow metabolism of methanol in the liver, and to a lesser extent in the blood, the symptoms of poisoning develop gradually depending on the increasing concentration of toxic metabolites in the organism (Yoo et al. 2016). Non-specific gastrointestinal symptoms including abdominal pain, nausea, vomiting, as well as other symptoms such as headache, general weakness, shortness of breath, and slight disturbances in the CNS function may occur as early as four hours after intoxication and may initially be misdiagnosed as ethanol poisoning (Grzybowski et al. 2015; Klein et al. 2017; Yang et al. 2005). Then, there is an asymptomatic period of about 10–12 h (Dorokhov et al. 2015). Usually, severe nervous system dysfunction is noticed 12–24 h after exposure to methanol (Seme et al. 2001), while visual symptoms usually appear within 12–48 h and occur in about 50% of cases (Grzybowski et al. 2015; Klein et al. 2017; Seme et al. 2001). In severe poisoning, disturbances of consciousness, memory loss, parkinsonism, severe cardiovascular symptoms, renal failure, rhabdomyolysis, coma, convulsions, and, consequently, death may occur (Choi et al. 2017; Klein et al. 2017; Yieh and Chou 2002). Lethal dose of methanol after oral ingestion is estimated to be 1.2 mL/kg (Noor et al. 2020); however, it has been shown that if the appropriate management is implemented promptly, poisoning with a much higher dose of nearly 7 mL/kg (920 mg/dL) can be successively treated (Martens et al. 1982).

## Methanol-induced optic neuropathy (Me-ION)

### Pathophysiology of Me-ION

Toxic exposure to methanol can result in damage to the optic nerve and to both the outer and inner retinal layers, while damage to the latter being generally more pronounced. Additionally, further parts of the visual system such as chiasm and optic tracts may also be affected (Grzybowski et al. 2015). In humans, even slight chronic exposure to low levels of methanol (0.87–1.0%) associated with severe malnutrition, especially in conjunction with a deficiency of cobalamin (vitamin B12) and folic acid—vitamins involved in the metabolism of methanol and its metabolites, can have dramatic consequences and result in the development of optic neuropathy. That was postulated in the leading hypothesis explaining the causes of the outbreak of the Cuban epidemic optical neuropathy (CEON) in 1992–1994, which affected 50,000 individuals, nearly 0.5% of the entire Cuban population (González-Quevedo et al. 2018; Hedges et al. 1997; Sadun 1998).

Previous human and animal studies have shown that systemic exposure to blood formic acid levels above 7 mM persist for more than 1 day may lead to long-term adverse visual effects (Eells et al. 1996). Importantly, animal studies have also shown that the distribution of toxic metabolites of methanol is characterized by varying intensity within the tissues. In rats, after 60 h after intoxication lasting 48 h with both low (4–6 mM) and high concentrations of methanol (8–15 mM), the highest concentration of formic acid was detected in the vitreous, retina, and blood, respectively. It is worth emphasizing that the concentration of formate in the optic nerve was five times lower than in the retina (Eells et al. 1996). While, in post-mortem human studies in methanol-poisoning victims, methanol accumulation in the vitreous was higher than in the aqueous humor (Benton and Calhoun 1953). Rodent model experiments also revealed that the distribution of methanol to tissues was independent of the degree of methanol exposure and was similar in rats intoxicated with both low (4–6 nM) and high (8–15 nM) concentrations (Eells et al. 1996).

### Molecular background of functional and histopathological changes in eye tissues in the course of Me-ION

**Fig. 2:**
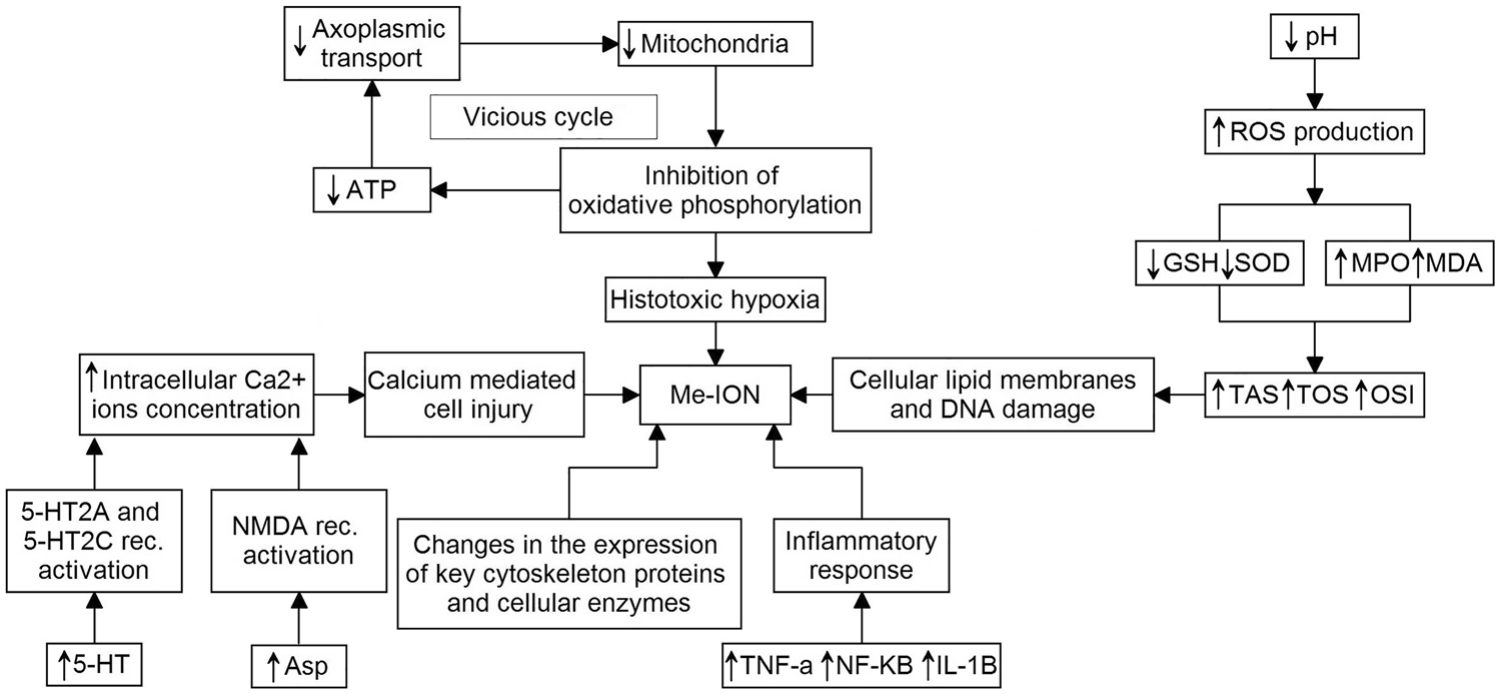
Mechanisms responsible for the molecular background of structural and functional changes in eye tissues in the course of methanol intoxication. Abbreviations: *5-HT* serotonin, *5-HT2A* serotonin 2A receptor, *5-HT2C* serotonin 2C receptor, *Asp* aspartame, *ATP* adenosine triphosphate, *GSH* glutathione, *IL-1β* interleukin 1β, *NF-ĸβ* nuclear factor-kappa B, *MDA* malondialdehyde, *Me-ION* methanol-induced optic neuropathy, *MPO* myeloperoxidase, *OSI* oxidative stress index, *ROS* reactive oxygen species, *SOD* superoxide dismutase, *TAS* total anti-oxidant status, *TNF-α* tumor necrosis factor-alpha, *TOS* total oxidant status.

The exact cause of the marked tendency to toxic damage to the optic nerve and the retina among the eye tissues by methanol and its metabolites is not fully understood; however, it is assumed to be due to the high energy dependence of the functional profile of these tissues, which is responsible for the much greater sensitivity to mitochondrial toxins such as formic acid. It is postulated that the main phenomenon determining the occurrence of pathological changes in the eye tissues in the course of methanol intoxication is mitochondrial dysfunction secondary to the binding of formic acid to the terminal enzyme of the respiratory chain—cytochrome c oxidase, or more precisely to its ferric heme iron (Dorokhov et al. 2015). This results in inhibition of the oxidative phosphorylation process with consequent tissue hypoxia and an increase in the production of reactive oxygen species (ROS) induced by a decrease in pH—these highly reactive molecules have the ability to damage key intracellular compounds including lipids and deoxyribonucleic acid (DNA) (El-Din et al. 2011). Inhibition of oxidative phosphorylation along with a decrease in pH results in histotoxic hypoxia and tissue damage (Fig. 2) (Masoud et al. 2016). In addition, recent investigations suggest that the inflammatory background also plays an important role in the development of pathophysiological changes in the eye tissues caused by the methanol and its metabolites (Ahiskali et al. 2019; Taşlı et al. 2018).

### The influence of methanol on mitochondrial function and oxidative stress parameters in the retina and optic nerve

In vitro studies with the use of formate showed that inhibition of ATP production in cultures of photoreceptor cells (661 W) and RPE (ARPE-19) increases with a decrease in blood pH; however, damage to photoreceptor mitochondria has also been found under neutral pH conditions after adding the sodium formate solution—these findings indicating the ability of formate to act as a mitochondrial toxin regardless of the pH value (Treichel et al. 2003). Animal studies based on a rat model showed the ability of retinal tissues to regain the balance of the mitochondrial energy function, but not anti-oxidant system after methanol poisoning—72 h after cessation of methanol intoxication; initially, reduced ATP and adenosine diphosphate (ADP) and increased adenosine monophosphate (AMP) concentrations did not differ significantly from the values observed in a control group; while the serum level of glutathione (GSH), a key endogenous molecule that determines the proper functioning of anti-oxidant mechanisms, was significantly decreased both during intoxication and after the 72 h observation period (Seme et al. 2001). The disturbance in the production of key molecules for cellular energy balance as one of the major mechanisms of tissue damage after methanol exposure is also supported by the results of the studies by Chen et al. where a significant reduction in the expression of ATP5A, an ATP synthase enzyme responsible for the production of ATP from ADP, was demonstrated in the retinal tissue exposed to methanol (Chen et al. 2012). Subsequent studies also showed changes in the expression of anti-oxidant defense components, as well as oxidative stress parameters. In the retinal tissue of rabbits exposed to methanol, a marked decrease in GSH concentration already after the first day reaching 60% of the initial value after 7 days along with a 70% increase in catalase serum level was found. In the same study, a change in the conformation of the rhodopsin alpha-helix, as well as the angle between rhodopsin and the cell membrane was observed, which as it can be assumed was the effect of the destructive effect of ROS on the lipid membranes structure (El-Din et al. 2011). The results of studies analyzing the effect of methanol intoxication on the levels of oxidative stress parameters in the optic nerve tissue in rats showed a decreased concentration of GSH, as well as superoxide dismutase (SOD), with a simultaneous increase in the level of myeloperoxidase (MPO) and malondialdehyde (MDA) and in the value of the index of total oxidative status (TOS), as well as the oxidative stress index (OSI), while the value of the total anti-oxidant status (TAS) was markedly reduced (Ahiskali et al. 2019; Icel et al. 2020; Taşlı et al. 2018). Interestingly, it has also been shown that intravenous ATP administration significantly reduces the negative influence of oxidative stress exerted by methanol and its metabolites on optic nerve tissue (Icel et al. 2020). To better elucidate the mechanisms responsible for the dramatic effect of methanol on mitochondrial function, Sadun developed the “mitochondrial catastrophe theory” presenting molecular changes secondary to mitochondrial damage as a vicious circle—according to this hypothesis, the decrease in the amount of ATP produced secondary to mitochondrial dysfunction results in insufficient supply of the Na + /K + ATPase pump with energy substrate, which in turn determines disruption of the axoplasmic transport of cellular organelles, including mitochondria along the axon, which leads to a decrease in the amount of available ATP at the target site (Sadun 1998).

### Methanol metabolism in retinal neurons: the role of peroxisomes in its retinotoxicity

Peroxisomes are small organelles that are especially abundant in the brain, liver, and adipose tissue. Their main functions include participation in α- and β-oxidation of fatty acids, lipid synthesis, and both the production and reduction of ROS (Daniele et al. 2019). It is presumed that the peroxisomes, especially abundant in Muller cells and RPE, due to the enzymes they contain: catalase and aldehyde dehydrogenase, may be involved in the local metabolism of methanol, which determines the formation of formaldehyde and formic acid (Garner et al. 1995; Glorieux and Calderon, 2017; Hayasaka et al. 2001; Icel et al. 2020; Sharma et al. 1999). This theory is supported by the results of studies in which the formate level in the retinal tissue was five times lower after systemic administration of formate compared to that measured after systemic administration of methanol at the corresponding dose (Garner et al. 1995). Therefore, this phenomenon may be an additional factor contributing to retinal damage.

### Formaldehyde toxicity

Although the most pronounced ocular tissue toxicity following methanol intoxication is attributed to formate, the role of formaldehyde toxicity should not be excluded. As mentioned, aldehyde may be produced locally in the retina as a result of methanol metabolism. Similarly, current animal studies have demonstrated the ability to metabolize methanol to formaldehyde within the CNS in primates after intracerebroventricular injection of a single dose of 200 µL of 5% methanol (Zhai et al. 2016). An experiment on the rat model showed abnormalities found in fundoscopy after 7 days, as well as 1 month after the injection of 0.1 and 1.0% formaldehyde solutions. Moreover, in the histopathological examination, pathological changes such as disorganization of the outer nuclear layer (ONL) and ganglion cell layer (GCL) structures were found after the injection of 0.1% formaldehyde. While the injection of 1% solution caused diffused disturbances in the structure of the retina. The administration of both mentioned concentrations of formaldehyde caused vacuolization of the optic nerve (Hayasaka et al. 2001).

### The role of serotonin (5-HT) and aspartame (Asp) in the course of Me-ION

It has been proven that apart from disturbing the process of energy transformations and the destructive influence of ROS, other mechanisms may also be responsible for the negative influence of methanol on the optic nerve and the retina. Increased concentrations of aspartame (Asp) in the optic nerve and serotonin (5-HT) in the retina were observed in rats chronically intoxicated with low doses of methanol (2 g/kg for 14 days) (González-Quevedo et al. 2002). Interestingly, elevated levels of Asp and glutamate (Glu) were also detected in CSF samples of CEON patients in whom chronic exposure to low levels of methanol along with folate deficiency was the most likely pathophysiological background (González-Quevedo et al. 2002). It is believed that the retinotoxicity of Asp is based on the mechanism of increased influx of calcium ions into cells due to the stimulation of N-methyl-D-aspartate (NMDA) receptors, the richness of which has been demonstrated within the retinal ganglion cell (RGC) layer from where the optic nerve axons originate (Shen et al. 2006). On the other hand, it is postulated that an increase in serotonin (5-HT) concentration results in the stimulation of 5-HT_2A_ and 5-HT_2C_ receptors in retinal neurons with a subsequent increase in intracellular calcium ion concentration, which, due to the disturbance of intracellular calcium homeostasis, promotes activation of signaling pathways responsible for retinal neurons death (González-Quevedo et al. 2002; Icel et al. 2020; Masson 2019).

### Inflammatory response secondary to methanol intoxication

Previous animal studies have shown that methanol intoxication can affect the function of the neuroimmune system and induce both specific and non-specific systemic immune responses, probably mainly by increasing oxidative stress and secondary changes in corticosterone levels resulting from dysfunction of the hypothalamic–pituitary axis (Moral et al. 2015; Parthasarathy et al. 2006, 2007). Examination of the optic nerve tissue of rats treated with the combination of methotrexate (MTX) and methanol showed higher expression of the final DNA degeneration product—8-hydroxy-2'-deoxyguanosine (8-OHdG), as well as molecules involved in the inflammatory response such as tumor necrosis factor-alpha (TNF-α), nuclear factor-kappa β (NF-kβ), and interleukin 1 beta (IL-1β), which suggests that the inflammatory background also plays an important role in the development of pathophysiological changes in the eye tissues caused by the methanol and its metabolites (Taşlı et al. 2018).

### Retinal proteins: changes in their expression secondary to methanol exposure

The results of current research on animal models indicate the possibility of much greater complexity of mechanisms determining cytotoxicity associated with methanol consumption. The toxic effect of methanol on the optic nerve and the retina may occur through the influence on the expression of key cytoskeleton proteins—actins and tubulins (beta-actin, beta-tubulin) conditioning the maintenance of the proper structure of cells, as well as cell signaling and regulating the process of apoptosis. In addition, changes in the expression of enzymes from the group of alcohol dehydrogenases: alcohol dehydrogenase (ADH), glyceraldehyde-3-phosphate dehydrogenase (GAPDH), long-chain acetyl-CoA dehydrogenase (VLCAD), family 5 member aldehyde dehydrogenase A1 (ALDH5A1), dehydrogenase 2 monophosphate-5' (IMPDH2), as well as proteins from the crystallin group and heat shock proteins (HSP) were also observed (Chen et al. 2012; Huang et al. 2011).

### Histopathological changes in eye tissues in the course of Me-ION

The number of histological studies of methanol toxicity to ocular tissues in humans is low, and the available results are limited to post-mortem studies and do not allow to fully assess which changes are a direct result of the action of methanol and its metabolites and which are post-mortem artifacts (Murray et al. 1991). Thus, a lot of valuable information was obtained using animal models in which mainly rodents were intoxicated. However, due to the much more efficient oxidation of formic acid in non-primates, due to the high activity of 10-CHO-THF, which in humans only reaches 26% of that observed in rodents, models using non-primates did not fully allow simulate the human conditions (Chen et al. 2012). Hence, to obtain a similar sensitivity to the toxicity of methanol and its metabolites as observed in humans, the animals were exposed to nitric oxide (NO), which inhibited the activity of methionine synthase (MS), and resulted in a secondary tetrahydrofolate deficiency, as well as inhibition of the activity of 10-CHO-THF—the decreased activities of these two enzymes lead to impaired metabolism of formate and its accumulation in the body (Chen et al. 2012). Another frequently used method is the introduction of a folate-deficient diet or the pharmacological induction of a folate deficiency by administering MTX (Eells et al. 1996).

### Animal histopathological studies

Chen et al. showed that in rats after intoxication with methanol, histopathological changes manifested mainly as mitochondrial damage are first found within the outer layers of the retina, starting from the photoreceptor layer, and spread further into the retinal inner nuclear layer (INL) (Chen et al. 2013). These observations are supported by the results of in vitro studies on the ocular cells where a decrease in ATP concentration after the administration of formate was detected earlier within the photoreceptor cell line (661 W) compared to the RPE cell line (ARPE-19). Moreover, also the histopathological changes were more pronounced in the 661 W cells, and the decrease in ATP concentration reflecting the disturbance of mitochondrial function at the examined time points correlated with higher levels of the cytotoxicity marker—lactate dehydrogenase (LDH) (Treichel et al. 2003). It is also supposed that the high content of polyunsaturated fatty acids (PUFAs), especially arachidonic acid (AA; 20:4n 6), in the photoreceptor layer may contribute to the damage of this layer by ROS in the lipid peroxidation process, mainly by its final product, malondialdehyde (MDA) (Ahiskali et al. 2019; Seme et al. 2001; Su et al. 2019). These findings suggest a greater structural and functional sensitivity of photoreceptors to formate-mediated toxic damage and indicate this retinal layer as the primary target of methanol-induced toxicity. Formic acid can penetrate the optic nerve from the CSF, as well as through the choroidal circulation, where it reaches a particularly high concentration in the retrolaminar region (Kavet and Nauss 1990). Histopathological analysis of the retinas and optic nerves of rats intoxicated with methanol showed pathological changes within the optic nerve constituting from the axons of retinal ganglion cells (RGCs), where pathological changes mainly concerned the prelaminar region and were expressed as axonal vacuolation and edema of the oligodendroglia and astrocytes manifested as myelin sheath damage (Eells et al. 1981, 2000; Galvez-Ruiz et al. 2015; Hayreh 1989). Vacuolisation of the axons of the laminar and post-laminar regions of the optic nerve head was also observed (Moschos et al. 2013; Rotenstreich et al. 1997). Examination of the optic nerves of Rhesus monkeys intoxicated with methanol revealed abnormalities mainly in the intraocular part of the optic nerve (Baumbach et al. 1977). In addition, axonal myelin sheath shrinkage was observed, causing axonal compression and honeycomb pattern in a histopathological examination—it is assumed that these findings result from the greater susceptibility of white matter composed of oligodendroglial cells to formate-mediated toxicity due to the lower content of cytochrome oxidase c compared to gray matter (Baumbach et al. 1977; Kavet and Nauss 1990). Thus, it is believed that the disruption of axoplasmic flow secondary to both ATP deficiency and mechanical axon compression by the contracted myelin sheaths caused by histotoxic edema of glial cells leads to stasis of the axoplasmic flow and plays a significant role in optic nerve damage (Baumbach et al. 1977). Ultrastructural changes at the axonal level observed by electron microscopy in addition to early degenerative changes included focal edema, axonal debris accumulation, fluid pocket formation, neurofibril breakdown, and axon-associated glial edema, which in some cases showed signs of gliosis. Mitochondria were characterized by a more elliptical shape, as well as larger and denser cristae or their disappearance (Sadun 1998; Seme et al. 2001). At the ultrastructural level, abnormalities in each of the layers of the retina can be observed. Evaluation of the methanol-exposed rats’ retinas revealed that within the RPE layer, vacuolization was dominant. Damage to the photoreceptors within the inner segments, where disorganization and swelling can be found, while the outer segment may become fragmented, and damage to the photoreceptor nuclei may also be present. Other studies have shown that vacuolization within the junction of the inner and outer segments of photoreceptors is also a distinctive finding (Eells et al. 1996; Rasheed et al. 2017; Seme et al. 1999, 2001). Typically, the mitochondria of the photoreceptor inner segments were damaged—swollen with enlarged or absent cristae; moreover, the number of these cell organelles may be reduced (Eells et al. 2003; Rasheed et al. 2017; Seme et al. 2001). In the outer nuclear layer (ONL), pathological changes manifested as edema and an increase in the space between the photoreceptor nuclei were noted; some of the nuclei were characterized by reduced size and apoptotic changes. Outer limiting membrane (OLM) may be interrupted or atrophic, while the outer plexiform layer (OPL) may have increased thickness (Rasheed et al. 2017; Zarenezehad et al. 2013). Irregular inner nuclear layer (INL) structure may also be present with apparent swelling and fragmentation of the nuclei. Edema may also occur within the inner plexiform layer (IPL); while RGCs may present apoptotic changes and vacuolation, and their number may be reduced (Rasheed et al. 2017).

### Human histopathological studies

The most characteristic changes reported by Sharpe et al. in four patients who died due to methanol poisoning were demyelinating changes in the retrolaminar region and swelling of the optic disc, appearing 2 days after intoxication, probably as a result of axoplasmic stasis (Sharpe et al. 1982). Damage to the myelin sheath in the retrolaminar region of the optic nerve was also noted by Naeser (Naeser 1988). In this case, retinal abnormalities including RGCs enlargement, moderate disturbances of the INL and ONL structure, as well as granulocytic infiltration of IPL were also observed (Naeser 1988). Postmortem examination of a patient who died in the course of CEON showed scarcity with concomitant chromatolysis of the RGCs nuclei with no changes in the photoreceptors and the peripheral retina. Additionally, reduced thickness of retinal nerve fiber layer (RNFL) within papillomacular bundle (PMB) was revealed. Interestingly, the reduced RNFL thickness in the PMB region was also observed in optical coherence tomography (OCT) examinations, and is presumed due to the increased sensitivity of small diameter mitochondria-rich nerve fibers to toxic formate damage (Nurieva et al. 2018). Histological analysis of six eyeballs enucleated for 6–18 h in victims of accidental methanol poisoning revealed degenerative changes in the photoreceptor layer and RGCs, as well as swelling of the optic disc and the peripapillary retina (Benton and Calhoun 1953).

### Assessment and diagnosis of Me-ION

Diagnosis in the case of suspicion of Me-ION is based on collecting a detailed medical history, especially focused on the possibility of consumption or contact with chemical substances containing methanol, laboratory tests, as well as detailed ophthalmological examination (Sharma et al. 2012).

### Laboratory tests

Laboratory tests revealing severe metabolic acidosis with the accompanying anion gap and plasma osmolality gap without any other clear cause should lead to the suspicion of methanol poisoning (Noor et al. 2020). Arterial blood pH has been shown to correlate with serum formate levels negatively, and a pH value below 7.2 indicates severe intoxication (Sharma et al. 2012). The concentration of methanol in the peripheral blood measured using liquid or gas chromatography exceeding 20 mg/dl allows to confirm the diagnosis of methanol poisoning (Sharma et al. 2012); however, due to the high cost, time, and low availability of this method in smaller centers, it may not always be used in the emergency conditions (Kraut 2016). Moreover, despite the usefulness of determining the serum concentration of methanol in the diagnosis of poisoning, it should be emphasized that this parameter does not correlate with the degree of intoxication of the organism and for this purpose, the assessment of arterial blood pH is much more valuable (Ma et al. 2019).

### Visual symptoms of Me-ION

Me-ION is usually bilateral, symmetrical, and in most cases presents a severe, rapidly progressive course (Sharma et al. 2012). During the presentation, patients may report eye pain located most often behind the eyeball, photophobia, and visual disturbances, which can manifest as either a slight reduction in visual acuity, up to the loss of vision, including a lack of light perception (Kraut 2016; Seme et al. 2001). On physical examination, eye redness and decreased pupil diameter or unresponsiveness of the pupils to light may be identified (Kraut 2016). Other symptoms, including the impaired contrast sensitivity, dyschromatopsia, blurred vision, diplopia, central or ceocentral scotoma with or without peripheral visual field defect, and more rarely, saccadic eye movements, as well as visual hallucinations may also be present (Ingemansson 1984; Sadun et al. 1994; Sharma et al. 1999). Importantly, in the case of chronic exposure to methanol leading to gradual intoxication, systemic and ocular symptoms may be initially limited and then recur with a stronger manifestation until a sudden crisis (Ma et al. 2019).

### Eye findings in patients with Me-ION

Slit-lamp examination of the anterior segment of the eye usually does not reveal significant abnormalities, although in some cases, fixed and dilated pupils with no response to light stimulation may be observed, this sign reflecting an unfavorable prognosis of final visual acuity and survival (Barceloux et al., 2002; Benton and Calhoun 1953). Intraocular pressure (IOP) is usually within the normal range (Klein et al. 2017; Sharma et al. 2012; Yang et al. 2005; Yoo et al. 2016). Analysis of the results of an ophthalmological examination after the outbreak of mass methanol poisoning in Atlanta showed the presence of fundus lesions in 87% of patients with initial vision loss and in nearly 100% of patients in whom continued follow-up examination showed a persistent decrease in visual acuity (Benton and Calhoun 1953). A fundus examination usually reveals bilateral hyperemia and edema of the optic disc, manifesting 6–24 h after methanol intoxication, with or without accompanying edema of the peripapillary RNFL, which may precede the onset of visual disturbances (Sharma et al. 2012; Yang et al. 2005; Yieh and Chou 2002). It is known that retinal edema is frequently observed along the major retinal vascular arches and may persist for 10–60 days (Benton and Calhoun 1953; Ingemansson 1984), then, after the resolution, transforms into retinal atrophy (Ingemansson 1984). In some cases, the characteristic wedge-shaped defects of RNFL may be present (Sadun et al. 1994). Macular edema along with the disappearance of macular reflex has also been reported (Ruedemann 1962). Changes in the optic disc appearance evolve with the course of intoxication from normal to swollen with dilated peripapillary and tortuous retinal vessels in acute, early phase (Cursiefen and Bergua 2002) to excavated and pale, in some cases glaucomatous-like with atrophy of the neuroretinal rim during the chronic phase, which is a sign of progressive degenerative changes (Grzybowski et al. 2015; Hayasaka et al. 2001; Moschos et al. 2013; Shin and Uhm 2011). These abnormalities are most likely developing based on acute RGC damage and progressive demyelination of nerve fibers, usually 30–60 days after intoxication (Benton and Calhoun 1953; Hayasaka et al. 2001; Yang et al. 2005). The atrophy of the optic disc may be more pronounced in the temporal segments (Nurieva et al. 2018). In sites of primary retinal edema, weakness and sheating of arterioles may be observed as a result of post-inflammatory changes (Dethlefs and Naraqi 1978).

### Imaging tests in the diagnostic process of Me-ION

The use of imaging tests plays an important role both at the initial stage of the diagnostic process as well as in monitoring the ophthalmic condition of patients affected by Me-ION. In the acute phase, OCT of the peripapillary region may show localized RNFL edema accompanied by the presence of intraretinal fluid (Fujihara et al. 2006), while in the late stage, OCT of the optic discs exhibits significant RNFL thinning that may progress up to 4 years after intoxication (Abrishami et al. 2011; Koehrer et al. 2011; Moschos et al. 2013; Nurieva et al. 2018; Shin and Uhm 2011). Macular OCT changes are characterized by a variable presentation—starting with no significant structural changes in the acute phase (Abrishami et al. 2011; Fujihara et al. 2006) by diffuse retinal thinning in the macular region observed 2 months after intoxication, to macular edema leading to flattening of the fovea with the presence of INL cysts observed 3 months after intoxication as a result of RGC loss (Fujihara et al. 2006). Interestingly, despite the particular susceptibility to damage to the photoreceptor layer observed in histopathological studies, the morphology of this layer in the reported OCT scans was normal (Koehrer et al. 2011). Fluorescein angiography (FA) initially reveals hyperfluorescence with late leakage in the peripapillary area; in addition, when retinal edema is present, this region may be characterized by late hypofluorescence (Fujihara et al. 2006); otherwise, multiple focal RPE detachments as an effect of intraretinal fluid accumulation have been observed (Ranjan et al. 2014). The classic finding in magnetic resonance imaging (MRI) is basal ganglion degeneration, typically with bilateral hemorrhagic or non-hemorrhagic putamen necrosis and edema or non-specific demyelinating lesions located in the deep white matter; whereas in toxic optic TONs of a different cause, these changes are usually not present (Grzybowski et al. 2015; Ma et al. 2019; Yang et al. 2005). Ma et al. described the bilateral enhancement of the signal of the optic nerves within the orbits and in the canal part as a frequent find in patients after methanol poisoning (Ma et al. 2019). At the same time, increased uptake of contrast in retrobulbar segments of optic nerves followed by slow diffusion of contrast has also been reported (Tanrivermis Sayit et al. 2016).

### Electrophysiological tests in the diagnosis of Me-ION

The importance of the application of electrophysiological tests such as electroretinography (ERG) and visual evoked potentials (VEPs) in the diagnostic process of Me-ION is indisputable. According to the results of animal studies, the abnormalities found in electrophysiological tests may precede the structural changes in the retina and optic nerve observed in fundus examination or imaging tests (Eells et al.^1996^; Seme et al. 1999).

### Visual evoked potentials (VEPs)

The results of VEPs usually show normal or less frequently prolonged latency, as well as abnormalities in waveform including weak or persistent no fixed waveforms, while the P100 amplitude in most cases is reduced (Grzybowski et al. 2015; Koehrer et al. 2011; Moschos et al. 2013). Interesting observations were made by McKellar et al., who observed a reduction in the P2 amplitude without significant changes in its latency, which is in contrast to the typical changes usually seen in optic neuropathies. Follow-up examination showed that the above-mentioned changes resolved 28 days after intoxication (McKellar et al. 1997). Another reported patient had mf-VEP abnormalities in area 0 (Moschos et al. 2013).

### Electroretinography (ERG)

Animal studies using ERG suggest that photoreceptors exhibit heterogeneous sensitivity to the toxic effects of methanol and its metabolites (Chen et al. 2013; Seme et al. 1999, 2001). In rats intoxicated with methanol, a partial recovery of the rod response to 15 Hz/510 nm stimulation was observed, while the UV-cone-mediated function showed no signs of regeneration in the ERG over the 72 h observation period (Seme et al. 1999). Similarly, the experiment of Chen et al. revealed the more pronounced cone damage in the F-ERG recording in rats after a 7 day period of methanol intoxication (Chen et al. 2013). On the other hand, Liu et al. showed no improvement in both scotopic and photopic recordings of ERG in rats performed on the third and seventh day after intoxication (Liu et al. 2016). In case reports involving human ERG recordings, a and b wave amplitude reduction is typically seen after both acute and chronic methanol exposure (Ingemansson 1984; McKellar et al. 1997; Ruedemann 1962), which proves abnormalities in the process of photoreceptor hyperpolarization, as well as depolarization disorders of Muller glial cells with disturbances in transmission between photoreceptors, bipolar cells, and RGCs, respectively (Eells et al. 1996; McKellar et al. 1997). Moreover, in severe cases, ERG may not be registered even several months after poisoning (Fujihara et al. 2006); interestingly, in the case described by McKellar et al. despite the initially observed abnormalities in the F-ERG, the scotopic and cone-mediated responses returned to normal on the 14th and 21st days after the poisoning, respectively (McKellar et al. 1997), which proves the ability to recovery photoreceptor function in humans, similarly as it was observed in animal studies. In rats subjected to long-term exposure to low concentrations (4–6 nM) of methanol, a significant reduction of the a and b amplitude in ERG was observed despite the absence of systemic markers of intoxication such as metabolic acidosis and histopathological changes in eye tissues (Eells et al. 1996). Importantly, in other human and animal studies, abnormalities in ERG were recorded at methanol concentrations that did not cause metabolic acidosis, disturbances in pupil reactivity, pathological fundus changes, and VEP abnormalities (Eells et al. 1996); these results show high specificity and greater sensitivity of ERG compared to the use of VEP in the diagnosis of patients with a suspicion of methanol poisoning (Plaziac et al. 2003). Moreover, the results of studies evaluating changes in ERG in intoxicated animals showed that the reduction in the amplitude of the b wave occurred faster and was more pronounced compared to the a wave, with the amplitude reduction of both waves being proportional to the increasing accumulation of formic acid in the serum (Eells et al. 1996). In animals exposed to lower concentrations of methanol (4–6 nM), deviations in ERG were recorded after 60 h, compared to 24 h in animals intoxicated with high concentrations of methanol (7–15 nM); this difference demonstrating a time-dependent relationship between the rate of intoxication and the occurrence of retinal dysfunction (Eells et al. 1996). Consistent results have also been obtained in other animal studies evaluating ERG after methanol exposure. In methanol-intoxicated rabbits, 1-week observation showed a linear decrease in the amplitude of the b wave reaching 35% in comparison to the initial value; amplitude of the a wave showed a clear tendency to stabilize (El-Din et al. 2011). In rats exposed on methanol at a dose of 1.5–4 g/kg (Garner et al. 1995; Lee et al. 1994; Murray et al. 1991; Plaziac et al. 2003) and inhaled methanol at a dose of 2000 ppm (Lee et al. 1994) also marked susceptibility of the b wave to the influence of methanol compared to the a wave was observed (Garner et al. 1995; Lee et al. 1994; Murray et al. 1991; Plaziac et al. 2003). In primates, exposure to methanol at a dose of 6 g/kg caused a reduction in the amplitude of the b wave and attenuation of the a wave (Potts et al. 1955); while in monkeys exposed to a lower dose of methanol, reaching 2–5 g/kg, no changes in the ERG were found (Blomstrand and Ingemannson 1984).

### Oscillatory potentials (OPs)

The ERG oscillatory potentials (OPs), also called low-voltage high-frequency oscillations, are localized on the ascending arm of the B wave and reflect low-amplitude impulses generated mainly by amacrine, bipolar, and interplexiform cells (Gauvin et al. 2016). Analysis of ERGs in methanol-intoxicated rats showed a reduction in summed amplitudes of wavelets (SOAP), as well as elapsed time (ET) delay which indicates damage to the receptor function of the retina. Similarly, the length of the interpeak interval (IPI) is 1–4, especially the IPI 2 increased with the length of the exposure period. Additionally, compared to non-intoxicated rats, a significant reduction in the amplitude of OS1, OS3, OS4, and OS5 was observed both after 3 and 7 days after methanol administration (Liu et al. 2016). Similarly, Plaziac et al. noted a significant reduction in OPs already 24 h after the exposure, which was maintained for the next 3 days (Plaziac et al. 2003). Interestingly, in both studies, the amplitude of OP2 was reduced to a lesser extent, while the most marked reduction concerned OP3 and OP4 (Liu et al. 2016; Plaziac et al. 2003). It is believed that short-latency OPs (OP1 and OP2) are generated mainly by cones, while rods play a major role in the formation of longer latency OPs (OP3, OP4, and OP5) (Liu et al. 2016).

### Application of electrophysiological tests in the research on new methods of Me-ION therapy

Seme et al. hypothesized that the lower sensitivity of the cone-mediated 15 Hz/510 nm response to methanol-induced damage demonstrated in the previous studies (Seme et al. 1999), and their impaired ability to recover after intoxication, suggests that they are less susceptible than rods to acute methanol damage; however, after marked disruption, cones do not show a tendency to regenerate (Seme et al. 2001). Current in vitro studies investigating the role of Muller cells as stem cells in the treatment of retinal injuries may shed new light on partial return of rod function after methanol exposure observed by Seme et al. (Seme et al. 2001). It has been shown that Muller cells are activated secondary to retinal damage and have the ability to migrate and proliferate at the site of injury mainly due to the activity of bioactive sphingolipid compounds (Hamon et al. 2016; Lenkowski and Raymond 2014; Vera et al. 2021). In vitro studies using human Muller cell lines have shown their ability to restore the function of both rods and RGCs (Hamon et al. 2016; Jayaram et al. 2014; Singhal et al. 2012). Despite the lack of in vivo human results, this hypothesis may explain this interesting phenomenon, and methods of regeneration of RGCs and photoreceptors may in the future constitute a new therapeutic option for patients affected by Me-ION.

### Treatment of Me-ION

Immediate initiation of treatment in Me-ION plays a key role in the prognosis of visual function after survival (Tanrivermis Sayit et al. 2016). Systemic treatment of methanol poisoning should be directed toward three main goals: first, limiting metabolic acidosis, second, inhibiting hepatic metabolism of methanol to its toxic metabolites, and third, facilitating the elimination of these compounds from the body (Barceloux et al. 2002; Sharma et al. 2012). These three steps in the treatment of methanol intoxication allow minimizing the toxic effects of methanol on tissues, including the eye tissues.

In the correction of metabolic acidosis, hemodialysis and intravenous bicarbonate administration are mainly used (Sharma et al. 2012). Hemodialysis allows for the rapid removal of methanol and formic acid from the body (Kraut 2016), while the increase in pH secondary to the use of bicarbonates reduces the entry of formic acid into the cells by increasing the ionization of methanol, and thus alleviating tissue damage (Garner et al. 1995; Tasli et al. 2018). The oxidation of methanol by alcohol dehydrogenase (ADH) is a key stage leading to the formation of its toxic metabolites in the body—hence, the first-line antidotes used in methanol poisoning are the competitive inhibitors of alcohol dehydrogenase—ethanol and fomepizole, also known as 4-methylpyrazole, preventing methanol metabolism to its toxic metabolites (Noor et al. 2020); their use allows to increase the ratio of unchanged methanol excreted from the body to metabolized methanol (Rasheed et al. 2017; Yieh and Chou 2002). Fomepizole has 500–1000 times stronger affinity for alcohol dehydrogenase compared to ethanol (Kraut 2016; Sharma et al. 2012); however, due to the much higher cost of fomepizole therapy, as well as a similar side effect profile and mortality rate, ethanol is a much more widely used antidote, especially in developing countries (Kraut 2016; Noor et al. 2020). It has been shown that methanol is oxidized in the body by alcohol dehydrogenase about ten times slower than ethanol (Rasheed et al. 2017), and the target serum ethanol concentration having an inhibitory effect on methanol conversion by ADH is 100–150 mg/dL (Kraut 2016). The tetrahydrofolate pathway plays a key role in the metabolism of formic acid to non-toxic metabolites—carbon dioxide and water (Abrishami et al. 2011). Thus, it is believed that the use of folinic acid may accelerate the metabolism of formic acid and, similarly to the use of hemodialysis, facilitate the elimination of methanol, and therefore shortening the time of its harmful effect on the body (Sharma et al. 2012). Dual therapy based on an ADH inhibitor and hemodialysis should be preferred when available, as this approach reduces the body’s exposure to methanol, preventing further complications, which contributes to lower treatment costs (Kraut 2016).

### Glucocorticosteroids

The initial phase of methanol-induced optic nerve damage is believed to resemble optic neuritis in its pathophysiological course (Permaisuari et al. 2019). Thus, the use of glucocorticosteroids (GCs) having the ability to down-regulate the expression of pro-inflammatory cytokines and up-regulate the expression of anti-inflammatory molecules, as well as neuroprotective and anti-demyelinating properties, has been implemented in Me-ION cases (Stunkel and Van Stavern 2018).

In the treatment of Me-ION, GCs are usually administered intravenously at a daily dose of 1 g methylprednisolone over 3–4 consecutive days, followed by oral prednisolone at a dose of 1 milligram per kilogram of body weight daily (Abrishami et al. 2011; Masoud et al. 2016; Sodhi et al. 2001). Abrishami et al. presented the results of an interventional case series involving six men poisoned with methanol after consuming homemade alcoholic beverages treated with 250 mg of intravenous methylprednisolone every 6 h for 3 days. Importantly, there was no additional treatment with hemodialysis, ethanol, or vitamins. The post-treatment examination showed an improvement in the mean BCVA 0.86 ± 0.08 vs 0.33 ± 0.18 and 0.93 ± 0.1 and 0.29 ± 0.2 for OD and OS, respectively (Abrishami et al., 2011). In other case series and case reports, a beneficial effect of steroid therapy on long-term visual sequelae has also been observed (Kowalski et al. 2019; Masoud et al. 2016; Sodhi et al. 2001). A collective statistical analysis including the results of published studies indicates the effectiveness of steroid therapy leading to the improvement of vision in more than 80% of eyes; however, it is worth emphasizing that the available studies were carried out on small groups without controls, on a case series or case reports (Permaisuari et al. 2019). Reports regarding late initiation of GCs therapy—more than 6 days after intoxication—show mixed results including no effect (Fujihara et al. 2006; Koehrer et al. 2011), as well as improvement of visual acuity (Rotenstreich et al. 1997). Shukla et al. in the group of 17 patients showed no association between the time of starting treatment with steroids, which reaching from 6th to 45th day, and the final visual acuity (Shukla et al. 2006). Therefore, further studies are needed to evaluate the efficacy of GCs use in Me-ION patients fully. According to the present knowledge, their role as an additional adjunctive treatment to conventional therapy appears to be beneficial and their inclusion in Me-ION patients in the absence of general contraindications should be considered.

### Erythropoietin

In recent years, the use of erythropoietin (EPO) due to its neuroprotective effect on glial cells, anti-inflammatory, anti-apoptotic, and anti-oxidant effects, as well as ability to improve the blood supply to damaged tissue has been recognized as a promising therapeutic method that can improve the visual acuity of patients suffering from different types of optic neuropathies (Pakravan et al. 2016). The beneficial effects of EPO in patients with toxic damage to the optic nerve secondary to methanol exposure are particularly presumed to be due to its ability to inhibit axon and RGC apoptosis, as well as the anti-oxidant activity exerted by an increase in the activity of two key enzymes in the anti-oxidant defense system—GSH and SOD (Pakravan et al. 2016). To date, one double-blind randomized clinical study and one case series investigating the effect of intravenous administration of EPO in Me-ION have been conducted, and both have shown promising results (Pakdel et al. 2018; Pakravan et al. 2016). Among two studies comparing the use of EPO as an adjunct to methylprednisone therapy, one revealed only transient visual acuity improvement in Me-ION patients treated with the combination of EPO and GCs. Better results in the group of patients in whom EPO have not been added were observed after follow-up period. However, in the second experiment including larger sample size, significant improvement in patients treated with EPO and GCs compared to therapy with EPO alone was noted (Pakravan et al. 2016; Zamani et al. 2018) (Table 1).

**Table 1:** Summary of studies that investigated the efficacy of erythropoietin (EPO) alone or in combination with glucocorticoids (GCs) in Me-ION patients

References	Sample size (eyes, *N*)	Intervention	Effects of the treatment	Explanatory comment
Pakdel (2019)	34 eyes (Study group: 20; Control group: 14)	Study (EPO) group: 20 000 IU of EPO in 100 mL 0.9% saline in 2 h for 3 days (i.v.) Control group: 100 mL 0.9% saline (i.v.)	BCVA improvement (≤ − 0.2 logMAR): 85% of the eyes in EPO group vs. 43% in placebo group [*p* = 0.01; CI (10–65%)] FBCVA: EPO group 1.84 vs. 2.79 logMAR in the control group (*p* = 0.012; 95% CI 0.23–1.74)	Phase-2 CT** (**NCT02376881**)** Double blind, randomized Criteria of eligibility: -Confirmed Me-ION -BCVA < 20/30 - < 3 weeks since methanol intoxication Primary outcome: BCVA at 16 week follow-up period
Zamani et al. (2018)	30 eyes (Study group: 20; Control group 10)	Study group: 10 000 IU of EPO twice a day for 3 days (s.c.) with 250 mg of MP four times per day for 3 days (p.o.) Control group: 250 mg of MP four times per day for 3 days (p.o.)	BCVA improvement (median (IQR) VA): Study group: 0.002 and 0.004 at discharge and follow-up, respectively (*p* = 0.4) Control group: 0.025 and 0.053 at discharge and follow-up, respectively (*p* = 0.19) FBCVA was significantly better in control group than in study group after follow-up period (*p* = 0.01) In the study group tendency to initial transient improvement in VA followed by further VA deterioration (with a mean 2 months period) has been noted No significant difference in the eye movement exam (*p* = 0.7), results of fundus examination (*p* = 0.055), and VEPs (*p* = 0.066)	Case–control study Criteria of eligibility: -Survivors with the presence of visual disturbances which were not improved after implementation of HD Follow-up period ranged from 9 months to 4.5 years 14 of 15 patients undergone HD, and received ethanol, folinic acid, as well as bicarbonate. One patient received ADH blocker and bicarbonate
Pakdel et al. (2018)	32 eyes	20 00 IU of EPO for 3 days (i.v.)	BCVA improvement: Median BCVA before treatment: light perception (3.90–0.60 logMAR) Median FBCVA (measured in the better-seeing eye): 1.00logMAR [(3.90–0.00 logMAR); *p* < 0.0001] Median FBCVA was significantly better in patients with VA of HM or better at admission (*p* = 0.025) No significant difference in FBCVA improvement depending on patient age or time between exposure to methanol and treatment with EPO in OD (*p* = 0.63) and OS (*p* = 0.80) No significant correlation between FBCVA and PRNFLT in the OD and OS	Prospective, noncomparative interventional case series Mean follow-up period: 7.5 months ± 55.8 Mean time period between exposure to methanol and treatment with EPO: 9.1 ± 5.56 days In 15 patients EPO course was repeated Criteria of eligibility: -Acute vision loss after methanol intoxication -Patients after detoxification treatment - < 3 weeks since methanol intoxication -Lack of VA improvement in the three preceding days -Lack of systemic disease -Value of anion gap in the serum < 11 mEq/L before admission Exclusion criteria: **-**Pregnancy or breastfeeding -Elevated BP -Positive history of TEEs or seizure -Hb level > 16 gm/dL -Positive history of eye disease or the presence of abnormalities during ophthalmological examination at admission -Treatment of ON with EPO or GCs in the past -Positive history of head injury in the last months
Pakravan et al. (2016)	44 eyes (Study group: 22; Control group 22)	Study group: 10 000 IU of EPO twice a day for 3 days + 500 mg of MP twice a day for 5 days with further 14 days of oral prednisolone 1 mg/kg/day Control group: 500 mg of MP twice a day for 5 days with further 14 days of oral prednisolone 1 mg/kg/day	BCVA improvement: EPO group: 2.93 ± 0.55 at presentation vs. 1.75 ± 1.16 logMAR (*p* < 0.001) after 3 months follow-up period Control group: 2.65 ± 0.68 vs. 2.19 ± 0.75 logMAR (*p* = 0.001) after 3 month follow-up period FBCVA was significantly better in EPO group compared to control group (*p* = 0.012) PRNFLT: EPO group: 131 ± 34 at presentation vs. 77 ± 26 after 3 months follow-up period Control group: 187 ± 24 at presentation vs. 53 ± 6 after 3 month follow-up period Final PRNFLT EPO group 77 ± 26 vs. 53 ± 6 in the control group (*p* < 0.001) FVFMD: EPO group 25.21 ± 6.83 vs. 23.25 ± 3.67 in the control group (*p* = 0.027)	Non-randomized interventional study Primary outcome: BCVA, PRNFLT and VFMD at 6 moths follow-up period Criteria of eligibility: - < 2 weeks since methanol intoxication Exclusion criteria: DM, uncontrolled HT, Hb level > 16 mg/dl

*10-CHO-THF* 10-formyltetrahydrofolate, *ADH* alcohol dehydrogenase, *ALDH* aldehyde dehydrogenase, *ALDH1A1* aldehyde dehydrogenase 1 family, member 1, *ALDH2* mitochondrial aldehyde dehydrogenase, *CYP2E1* cytochrome P450 Family 2 Subfamily E Member 1, *NAD* nicotinamide adenine dinucleotide, *NADH* reduced form of nicotinamide adenine dinucleotide, *NADPH* nicotinamide adenine dinucleotide phosphate

### Photobiomodulation

Photobiomodulation (PB), also known as low-intensity light therapy, involves applying light with a wavelength of 630–1000 nm and a far-red to near-infrared (NIR) spectrum emitted by low-level lasers (LLLs) or light-emitting diodes (LEDs) to modulate specific cell functions (Desmet et al. 2006). Although the exact mechanism of the beneficial effect of this method has not been fully understood, it is assumed that PB improves mitochondrial function by stimulating the activity of cytochrome c oxidase c complex IV, leading to an increase in ATP synthesis (Yu-Wai-Man et al. 2014).

PB has been shown to have neuroprotective properties, promote the wound-healing process, reduce heart damage after ischemia, and may also be useful in patients with congenital (e.g., Leber's congenital optic neuropathy and autosomal dominant optic atrophy), as well as acquired toxic-mediated injury of the optic nerve and retina, including the Me-ION (Desai et al. 2013). The animal study showed a protective effect on the photoreceptors function and retinal structure after three 144 s sessions of NIR-LED light with a wavelength of 670 nm applied at 5, 25, and 50 h after intoxication with methanol (Eells et al. 2003). In this study group, after the use of high light intensity PB, a partial recovery of the function of UV-cones, M-cones, and rods was observed in rats. In addition, in the LED-treated group, no histopathological changes of the retina were shown either in light microscopy or at the ultrastructural level, while in the group without PB application, clear pathological changes were observed (Eells et al. 2003).

### Anti-oxidants

In recent years, promising results of animal studies investigating the effect of using compounds with anti-oxidant properties such as taxifolin (Ahiskali et al. 2019), ATP (Icel et al. 2020), as well as rutin (Taşlı et al. 2018) have been published. The above studies showed a significant effect of these compounds on the alleviation of methanol-induced oxidative stress in the nerve tissue of rats. Another study showed the protective potential of 4-hydroxy-2,2,6,6-tetramethylpiperidinyl-1-oxyl (TEMPOL)—a chemical compound having SOD mimetic properties on RGCs exposed on methanol (Setiohadji et al. 2018). However, further research is needed to evaluate the efficacy of these antioxidants in humans fully. Additionally, in some cases, the vitamin B group (e.g., vitamin B1, B6, and B12) has been used (Rotenstreich et al. 1997); but there are no studies in which their protective potential to ocular tissues in patients with Me-ION has been investigated.

## Prognosis of patients with Me-ION

### Ocular prognosis

In most patients, visual disturbances usually resolve within 2–3 weeks after intoxication; however, persistent visual sequelae are estimated to affect more than 33–40% of patients (Nurieva et al. 2018). Moreover, newly emerging or initially undiagnosed ailments may be found many months after intoxication (Nurieva et al. 2018; Paasma et al. 2007). To date, the results of several studies assessing the prognosis of visual complications in patients after methanol poisoning have been published. A 3 month follow-up of 24 patients from Port Moresby in Papua New Guinea showed that the presence of metabolic acidosis on admission and the amount of consumed methanol positively correlated with the occurrence of persistent visual impairment (Dethlefs and Naraqi 1978). Sullivan-Mee and Solis hypothesized that the pupil reactivity in the initial examination may provide important prognostic information for the further visual function, and their abnormal response is associated with a worse prognosis and a greater risk of permanent visual abnormalities (Sullivan-Mee and Solis 1998). This assumption was indirectly confirmed by the results of a study evaluating the presence of long-term visual sequelae in a group of 122 individuals after methanol poisoning, where diminished pupil reactivity and abnormalities in the fundoscopy during the initial examination were observed significantly more often in patients with arterial blood pH value lower than 7.2 measured on admission, and follow-up showed less improvement and lower visual acuity after 3 months (Desai et al. 2013).

Interesting results were obtained in the studies of the population affected by Me-ION as a result of massive poisoning in the Czech Republic in 2012, including 139 cases, and more than 50 deaths (Nurieva et al. 2018; Zakharov et al. 2014). Zakharov et al. showed that despite the presence of visual symptoms in 14% of patients at discharge, the frequency of the occurrence of persistent visual sequela in the 3–8 month period of follow-up increased to 40%, and blindness affected 8% of patients (Zakharov et al. 2014). Risk factors associated with a higher probability of persistent visual sequelae were visual disturbances and coma revealed on admission, while concomitant ethanol consumption significantly reduced the likelihood of their occurrence. In addition to the analysis of the correlation between clinical parameters, the assessment of RNFL thickness using OCT and the evaluation of VEP components—P1 latency—as an indicator of the demyelination and remyelination process, as well as the N1P1 amplitude—as an indicator of RGCs damage were used to determine the prognosis in patients with Me-ION. Pathological changes in RNFL and abnormalities observed in VEP recordings were detected in a follow-up examination 3–8 months after discharge in 38% and 40% of survivors after methanol poisoning, respectively, with 28% borderline results in RNFL, and 18% borderline results in VEP (Zakharov et al. 2015). It was shown that the presence of serum ethanol on admission was associated with a lower probability of damage to the axons of the optic nerve and a higher RNFL thickness measured during the observation period. Moreover, RNFL thinning and abnormalities in VEP showed a mutual correlation, which reached statistical significance in the second, but not in the fourth year after discharge (Nurieva et al. 2018; Zakharov et al. 2015). In 22% of patients without visual symptoms at discharge, RNFL thinning and VEP abnormalities were observed after 3–8 months (Zakharov et al. 2015). P1 latency abnormalities were observed on admission in 50% of patients; however, during the surveillance period, a significant reduction in its duration was observed in cases where the initial P1 latency length corresponded to mild or moderate damage to the myelin sheath, which proves the ability to remyelinate nerve fibers after acute methanol damage (Nurieva et al. 2016). Significantly in 2 years of observation, chronic alcohol abuse was associated with worse effectiveness of the remyelination process, while beneficial factors for the regeneration of myelin were both lower serum concentrations of carbohydrate-deficient transferrin and methanol, as well as higher blood pH value during the presentation (Nurieva et al. 2016). Additionally, in older individuals, longer P1 latency during 2 year follow-up was observed, which indicates a lower efficiency of the optic nerve axon remyelination process (Nurieva et al. 2016). Moreover, a significant association between the risk of thinning RNFL, and the value of arterial blood pH measured at presentation, as well as the abnormal morphological changes in the CNS on MRI, were also found (Nurieva et al. 2016). The reduced P1 latency observed in the VEP returned to normal during the 2 year follow-up period, probably due to remyelination process of the nerve fibers; therefore, the initially observed relationship between P1 latency extension and RNFL thinning became insignificant at the end of the observation period (Nurieva et al. 2016); however, the reverse trend was observed for the N1P1 amplitude, where along with the duration of the observation period, the correlation between the height of the N1P1 amplitude and the RNFL thickness was significant—in patients with lower N1P1 amplitude, a thinner RNFL was observed. Interestingly, the extent of the initial RGC damage significantly influenced the course of chronic thickness loss of RNFL (Nurieva et al. 2016). During the 4 year follow-up period, a significant thinning of RNFL, especially in the temporal quadrant, was present in repeated OCT studies in 31% of patients (Nurieva et al. 2018). The factor that most strongly correlated with decreasing RNFL thickness in subsequent years was lower arterial blood pH at admission; however, for a higher serum creatinine concentration and a lower serum ethanol concentration, a significant relationship was also revealed (Nurieva et al. 2018). Interestingly, it was shown that patients with ApoE polymorphism showed a more pronounced tendency to develop RNFL thinning and prolongation of initial P1 latency compared to patients without this polymorphism (Nurieva et al. 2018). In addition, a clear preference to impaired visual function, as well as abnormalities in MRI of the brain, the presence of necrosis and hemorrhages, was found in this group (Nurieva et al. 2018).

### General prognosis

Many factors that adversely affect the survival prognosis in methanol-intoxicated patients have been identified, including high serum lactate and potassium levels, lower peripheral blood pH, lower serum bicarbonate levels, and higher osmolar and anion gaps at presentation (Noor et al. 2020). Additionally, higher mortality was also found in patients with more prolonged exposure to methanol, as well as in the older age (Ma et al. 2019). Moreover, coma upon admission to the hospital and lack of simultaneous ethanol consumption were also negative factors (Zakharov et al. 2014). Therefore, due to the multiplicity and diversity of prognostic factors, mortality in the acute phase of methanol intoxication ranges from 18 to 44% (Noor et al. 2020).

Studies investigating 6 year mortality after methanol poisoning in patients suffering from Me-ION in both the Czech (Zakharov et al. 2020) and Estonian (Paasma et al. 2009) populations showed 18% and 30% death rate values, respectively. What is worth emphasizing, in the study by Zakharov et al., 47% of deaths were associated with the occurrence of neoplastic, which was probably related to the carcinogenic effect of acute exposure to methanol and its metabolites, especially formaldehyde, and secondary, significantly increased oxidative stress (Zakharov et al. 2020). In the Estonian population, characterized by a similar age structure, but with a higher ratio of females to males compared to the Czech population (31/69 vs. 20/80%), it was shown that the dominant cause of death in the studied group was alcohol intoxication, which occurred in 27% of patients (Paasma et al. 2009). Thus, the results of the above observations show that survivors should be subject to comprehensive supervision and multidisciplinary medical care in further years after Me-ION.

## Conclusions

Although, Me-ION has been known for many years and is not a rare problem for clinicians and researchers worldwide. The mechanisms determining the occurrence of pathological changes in its course have not been fully understood, which is the reason for the lack of available effective treatment methods that would protect patients against dramatic visual consequences of methanol poisoning. The mechanism of damage to the structures of the visual system, in particular, the optic nerve and the retina, is multifactorial; therefore it is difficult to develop targeted therapeutic methods of treatment. Patients affected by Me-ION require comprehensive medical care also in the years following poisoning due to adverse health consequences leading to a significant deterioration in the quality of life. Therefore, intensive efforts to reduce the rate of methanol poisoning based on both social education and the development of new therapies and strategies for managing Me-ION patients should be continued.
